# Pediatric orbital solitary fibrous tumor/hemangiopericytoma presenting with isolated eyelid edema: a case report

**DOI:** 10.3389/fped.2026.1884656

**Published:** 2026-08-26

**Authors:** Cheng Gai, Lijuan Zhang, Xiaofei Zhao, Bing Wang

**Affiliations:** Department of Ophthalmology, Shandong Provincial Hospital Affiliated to Shandong First Medical University, Jinan, Shandong, China

**Keywords:** child, hemangiopericytoma, magnetic resonance imaging, orbit, solitary fibrous tumor

## Abstract

**Background:**

Orbital solitary fibrous tumor/hemangiopericytoma (SFT/HPC) is an exceedingly rare mesenchymal neoplasm in the pediatric population. While typical orbital tumors present with proptosis or mass effect, the clinical picture can be deceptively indolent, leading to diagnostic delay.

**Case presentation:**

A 15-year-old male patient presented with a 2-year history of progressive right upper eyelid edema that had repeatedly been misdiagnosed as conjunctivitis and visual fatigue. Notably, there was an absence of proptosis, visual decline, or ocular motility restriction. Magnetic resonance imaging revealed an extraconal mass posterior to the right globe. The patient underwent lateral orbitotomy with *en bloc* resection of a 4.5 cm × 3.5 cm × 2.5 cm dark-red, pseudocapsulated tumor. Histopathology and immunohistochemistry (CD34+, CD31–, S-100–, SMA–, and Ki-67 3%) confirmed the diagnosis of SFT/HPC. Given the tumor size and the potential risk of recurrence, adjuvant low-dose radiotherapy was administered after a multidisciplinary discussion.

**Conclusion:**

This case underscores that persistent, asymmetric eyelid edema in a child—even in the absence of classic orbital signs—warrants dedicated orbital imaging to exclude space-occupying lesions. Complete surgical resection via lateral orbitotomy provides the definitive diagnosis and effective local control for orbital SFT/HPC.

## 1 Introduction

Orbital solitary fibrous tumor/hemangiopericytoma (SFT/HPC) is a rare soft tissue tumor in clinical practice, and its occurrence in the orbit during childhood is even rarer, making it prone to missed diagnosis and misdiagnosis (1). According to the current World Health Organization (WHO) classification of soft tissue tumors, hemangiopericytoma is now considered within the solitary fibrous tumor (SFT) spectrum (2). This article reports the diagnosis and treatment of a pediatric orbital SFT/HPC in our department, reviews relevant literature, and discusses the source, clinical manifestations, diagnosis, and treatment of this disease.

## 2 Case presentation

**Figure 1:**
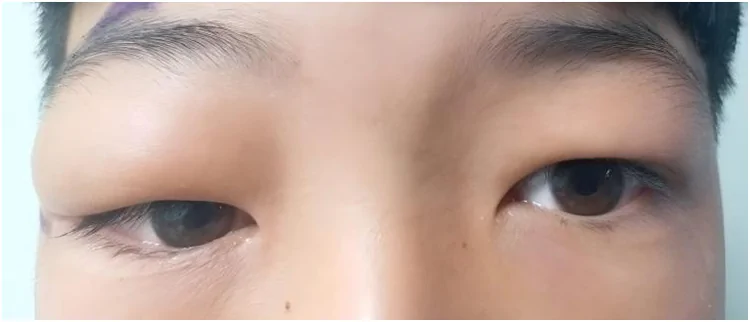
Preoperative image: the patient has swelling of the upper eyelid of the right eye without obvious eyeball protrusion. The left eye appears normal.

**Figure 2:**
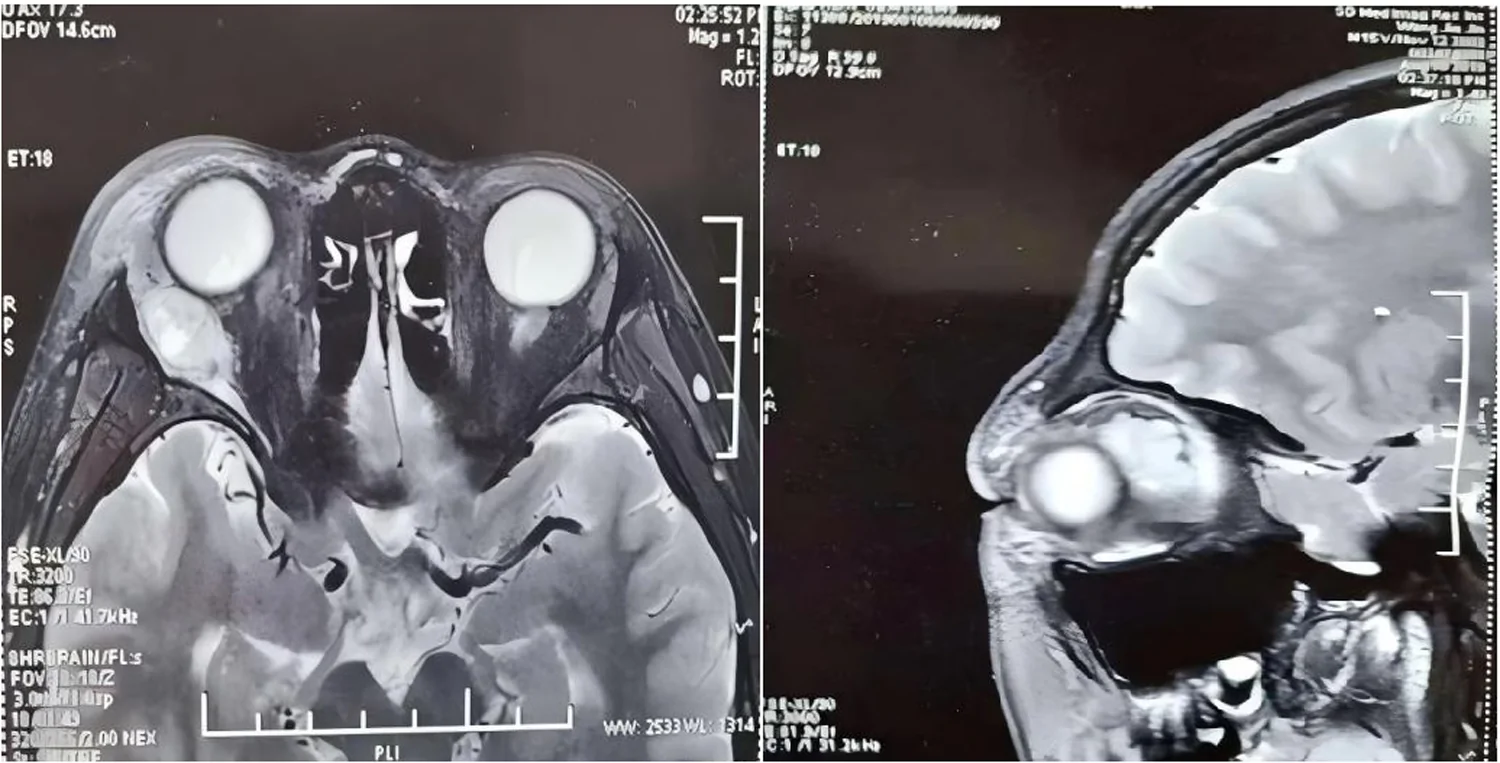
Axial and sagittal fat-saturated T2-weighted orbital MRI shows an extraconal hyperintense soft tissue mass in the right orbit located in the lacrimal fossa and adjacent to the lateral rectus muscle, measuring approximately 2.4 cm × 2.3 cm × 1.4 cm. The left orbital soft tissues appear normal within the imaging field.

**Figure 3:**
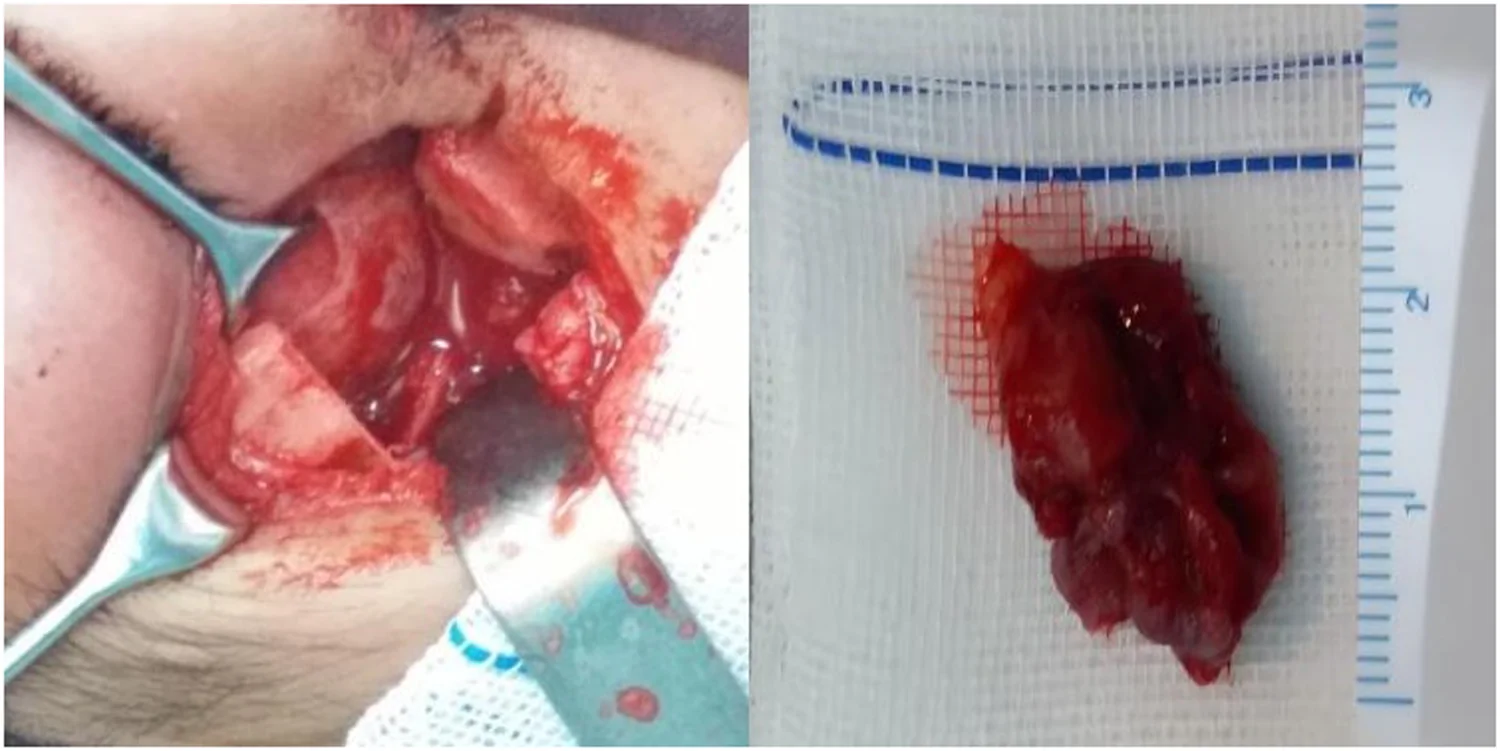
Intraoperative image: after the lateral orbital opening, the tumor has a clear boundary, is a dark-red color, and is covered by a pseudocapsule. The tumor is generally solid, dark-red, nodular, without an obvious capsule, and rich in blood vessels.

**Figure 4:**
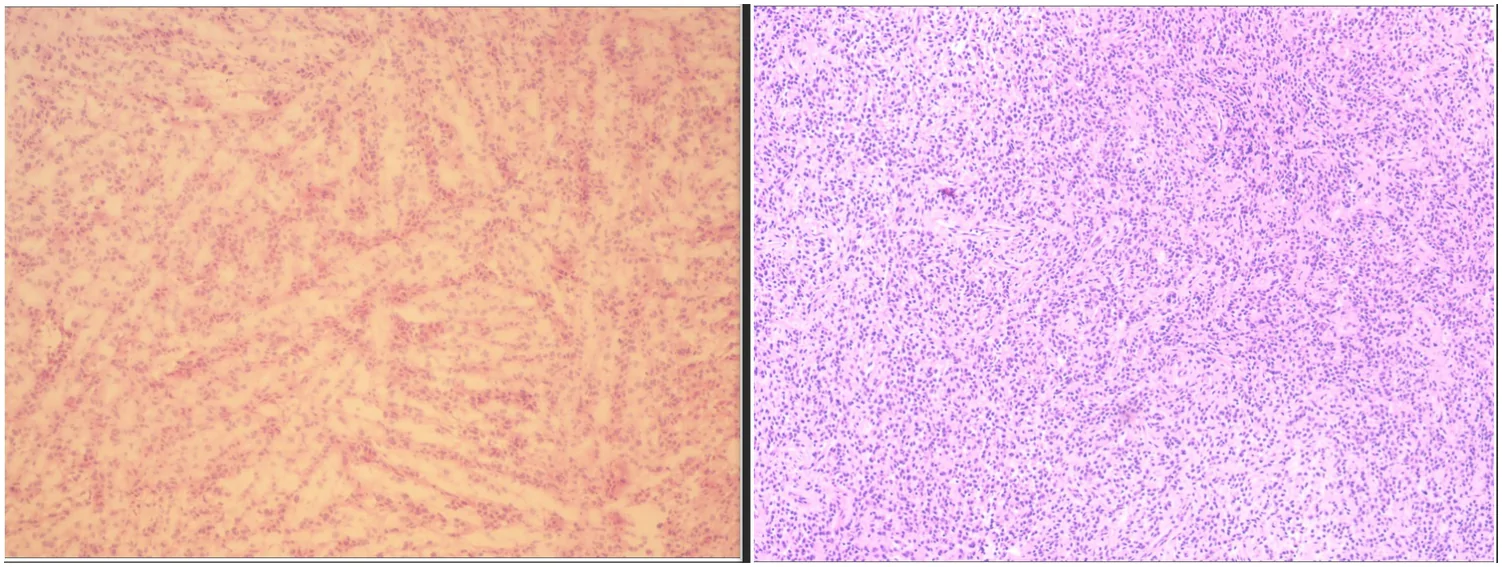
Pathology report: the histopathological examination of the right orbital tumor shows a small round cell proliferative lesion, and immunohistochemistry demonstrates CD34 positivity with negative staining for CD31, S-100, SMA, CK, and SOX10, along with scattered Caldesmon positivity and a Ki-67 index of 3%.

**Figure 5:**
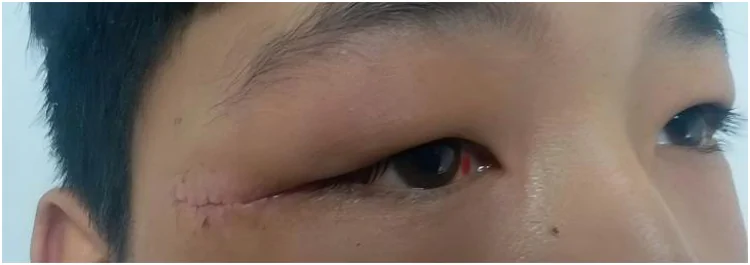
The swelling of the upper eyelid of the right eye is reduced, and eye movement is normal 1 week after surgery.

A 15-year-old male patient presented with a 2-year history of right upper eyelid edema that had worsened with associated lower eyelid edema over the preceding month (Figure 1). Visual acuity was 1.0 bilaterally, with intraocular pressures of 18 mmHg in the right eye and 13 mmHg in the left. The right eye showed moderate upper eyelid swelling, mild lower eyelid swelling, and no palpable mass in the lacrimal gland area. The conjunctiva was not congested, the cornea was clear, and the anterior segment and fundus were unremarkable; ocular motility was full without restriction. The left eye was essentially normal. The symptoms began insidiously without an identifiable cause more than 2 years before presentation. The patient had been previously treated for presumed conjunctivitis and visual fatigue before orbital magnetic resonance imaging (MRI) revealed a well-defined extraconal mass located in the right lateral orbital space, posterior to the globe (Figure 2). The lesion demonstrated hypointensity on T1-weighted imaging (T1WI) and hyperintensity on T2-weighted imaging (T2WI). The mass was closely associated with the lateral rectus muscle, but it showed a pushing border without clear invasion. No bone erosion or remodeling was noted. No diffusion-weighted imaging (DWI) sequences were available at the time of initial imaging. The patient was subsequently hospitalized for surgery. On admission, the general physical examination was unremarkable; computed tomography and ultrasonographic screening of other body regions revealed no additional space-occupying lesions. After completion of the preoperative systemic examination, the tumor was removed under general anesthesia through an orbital opening on the lateral side of the right eye. During the surgery, the tumor was found in the lateral orbital space in close proximity to the lateral rectus muscle, presenting a pushing rather than an invasive state. There was a clear boundary between the tumor and the muscle. A lateral orbitotomy with bone flap removal was performed to achieve adequate exposure; the bone flap was repositioned at closure. The mass was removed *en bloc* without violation of the pseudocapsule. The lateral rectus muscle was protected with a muscle hook and remained intact throughout the procedure. No obvious damage was observed. The surface of the mass was covered with a thin pseudocapsule, measuring approximately 4.5 cm × 3.5 cm × 2.5 cm (Figure 3). The intraoperative evaluation suggested the margins were negative, but no frozen section was sent for pathological assessment. The histopathological examination of the resected tumor revealed a solid, dark-red, nodular mass composed of dense, uniform spindle cells without specific arrangement. A characteristic “staghorn” (antler-like) vascular pattern was identified. Immunohistochemistry demonstrated CD34 positivity with negative staining for CD31, S-100, SMA, CK, and SOX10, along with scattered Caldesmon positivity and a Ki-67 index of 3%, all consistent with the histomorphology of a solitary fibrous tumor (Figure 4). Of note, STAT6 immunohistochemistry and NAB2-STAT6 fusion molecular testing were not performed due to technical unavailability at our institution during the diagnostic period. A multidisciplinary team discussion involving ophthalmology, radiation oncology, and pathology was held. Although the resection appeared complete, adjuvant radiotherapy was recommended because of the relatively large tumor size (4.5 cm) and the known potential for late recurrence in SFT/HPC, even after complete excision (3). The radiation oncology department formulated an individualized low-dose regimen (total dose of 4,400 cGy in 22 fractions, 200 cGy per fraction, delivered to the tumor bed with a 5 mm margin), which was initiated 3 weeks postoperatively. After 6 months of follow-up, eyelid edema decreased, no tumor recurrence was observed in the eyes, and vision and eye movement were both normal (Figure 5).

## 3 Discussion

Orbital SFT/HPC is a rare mesenchymal tumor in clinical practice (3). To date, only a limited number of pediatric orbital cases have been reported globally, and the condition is easily missed or misdiagnosed due to its indolent presentation.

The tumor was first named hemangiopericytoma by Stout and Murray (4). The majority of SFTs/HPCs are solitary, and the etiology remains unclear. This disease can occur in any part of the body, mainly in the central nervous system, skeletal muscle system, and retroperitoneum, with rare orbital involvement. Ribeiro et al. (5) reported a 12-year-old girl with a 6-year history of a large orbital mass that was completely excised. Someda et al. reported a 12-year-old patient with a CD34-negative orbital SFT (with nuclear STAT6 expression ) (6). Malkawi et al. (7) reported a 13-month-old child with an orbital SFT, the youngest documented patient with this tumor. Vu et al. (8) reported a case of a recurrence in a 12-year-old boy. Compared to these previously reported cases, our patient presented with isolated eyelid swelling for 2 years, without eye bulging, vision problems, or movement issues. This atypical and slow-progressing presentation led to multiple misdiagnoses. The well-circumscribed tumor displaced the lateral rectus muscle rather than invading it, which may partly explain the prolonged indolent course and delayed diagnosis.

The differential diagnosis of pediatric orbital masses is broad and includes vascular tumors (e.g., cavernous hematoma or venous malformations), fibrous masochistic lesions, rhabdomyosarcoma, schwannoma, non-proliferative disorders, and metastatic retinoblastoma. In this case, the well-circumscribed, pushing border on MRI, the absence of bone destruction, and the characteristic CD34+/CD31-/S100- immunophenotype helped exclude rhabdomyosarcoma (which typically shows diffuse S100 negativity but muscle marker positivity), schwannoma (S100+), and vascular malformations (CD31+ and ERG+). No pathogenic MRI findings specific to orbital SFT/HPC have been established. MRI findings in intracranial and other body sites are described as solitary, irregular, and ambulatory masses with heterogeneous signals. T1WI usually shows isointense or slightly hypointense signals, while T2WI shows hyperintense signals (9). Necrotic cystic degeneration may show lower T1 and higher T2 signals, and enhancement is typically heterogeneous. The MRI findings in this case are largely consistent with the literature, although DWI and apparent diffusion coefficient mapping were not available.

The diagnosis of SFT/HPC relies on histopathological and immunohistochemical evaluation. Immunohistochemistry typically shows positivity for CD34 and vimentin, with negativity for Epithelial Membrane Antigen (EMA), S-100, and CD31 (10). We acknowledge that in the current diagnostic era, nuclear STAT6 expression and/or NAB2-STAT6 fusion testing are considered essential for confirming the diagnosis of SFT/HPC. STAT6 immunohistochemistry has been shown to be a highly sensitive and specific marker for SFT, with nuclear STAT6 expression being a distinguishing feature that helps differentiate SFT from histological mimickers (11). These tests were unavailable at our institution, which constitutes a diagnostic limitation. However, the diagnosis in this case was established based on the following: (1) the characteristic histomorphological features, including the staghorn vascular pattern and uniform spindle cell proliferation (12); (2) the classic immunohistochemical profile (CD34+, CD31−, S-100−, and SMA−); and (3) the systematic exclusion of other entities in the differential diagnosis. We recommend that future cases undergo STAT6 immunohistochemistry and molecular testing whenever feasible to strengthen diagnostic certainty.

Recent studies have consistently defined SFT/HPC as a borderline malignant tumor, with local recurrence rates ranging from 13% to 40%, and distant metastases to the lung, bone, and liver (13, 14). Since SFT/HPC has a pseudocapsule but may have microscopic extensions, complete resection is considered the mainstay of treatment. The lateral orbitotomy approach adopted in this case allowed for complete and direct tumor removal, minimizing the risk of partial resection and tumor spillage.

The cautious decision to administer adjuvant radiotherapy in this pediatric patient was made despite an apparently complete resection and a low Ki-67 index (3%) due to the tumor size (4.5 cm) and the well-documented potential for late recurrence—including cases reported more than 30 years after initial surgery (15). Furthermore, some retrospective studies have shown that compared to surgery alone, adding radiotherapy after surgery can reduce the chance of recurrence (16); therefore, after a multidisciplinary discussion, a consensus was reached with the patient's family. The regimen used (4,400 cGy in 22 fractions) was a risk-adapted, individualized protocol, not standard pediatric radiotherapy, and was delivered with modern conformal techniques to minimize exposure to adjacent critical structures. Long-term follow-up is planned to monitor both recurrence and radiation-related complications.

## 4 Conclusion

This case underscores the importance of considering orbital neoplasms in the differential diagnosis of persistent, unilateral eyelid edema in children, even in the absence of proptosis or visual symptoms. Lateral orbitotomy allows for complete tumor resection with low morbidity, and adjuvant radiotherapy may be considered on an individualized basis for high-risk features.

## Data Availability

The original contributions presented in the study are included in the article/Supplementary Material, further inquiries can be directed to the corresponding author.
